# Phosphorylated IGFBP-1 in predicting successful vaginal delivery in post-term pregnancy

**DOI:** 10.1007/s00404-014-3577-x

**Published:** 2014-12-19

**Authors:** Katarzyna Kosinska-Kaczynska, Dorota Bomba-Opon, Katarzyna Bobrowska, Szymon Kozlowski, Robert Brawura-Biskupski-Samaha, Iwona Szymusik, Piotr Wegrzyn, Miroslaw Wielgos

**Affiliations:** 1st Department of Obstetrics and Gynecology, Medical University of Warsaw, Pl. Starynkiewicza 1/3, 02-015 Warsaw, Poland

**Keywords:** Insulin-like growth factor binding protein-1, Post-term pregnancy, Vaginal delivery, Bishop score, Cervical length

## Abstract

**Purpose:**

To estimate whether phosphorylated IGFBP-1 (phIGFBP-1) in cervical secretion in term and post-term pregnancies can predict spontaneous onset of labor or vaginal delivery.

**Methods:**

A prospective cohort study of 167 women in singleton term and post-term pregnancies, was conducted at 1st Department of Obstetrics and Gynecology, Medical University of Warsaw, between 2013 and 2014. phIGFBP-1 test (Actim Partus Medix Biochemica), ultrasound cervix assessment and Bishop score were analyzed in the study group. Spontaneous onset of labor was the primary and vaginal delivery was the secondary outcome.

**Results:**

In 32.5 % of patients, spontaneous uterine contractions appeared. 67.5 % of women delivered vaginally, 32.5 % had cesarean section. phIGFBP-1 test predicted spontaneous onset of labor (sensitivity 0.69, specificity of 0.42) and successful vaginal delivery (0.67, 0.48). In the prediction of spontaneous delivery onset ultrasound cervical assessment and phIBFBP-1 had comparable sensitivity and in the prediction of successful vaginal birth all three tests had comparable sensitivity. The time from preinduction to spontaneous onset of delivery was significantly shorter in women with positive phIGFBP-1 test (13.65 ± 6.7 vs 20.75 ± 2.6 h; *p* = 0.006).

**Conclusion:**

A test for phIGFBP1 presence might be an additional tool for predicting both spontaneous onset of labor and successful vaginal delivery in post-term pregnancies.

## Introduction

Insulin-like growth factors I and II (IGF I and II) are well-known agents controlling cell and tissue growth and metabolism. Han et al. and Irwin et al. [1, 2] have shown that IGF-I and -II are synthesized in placental trophoblast and fetal membranes from 6th week of gestation. In biological fluids IGFs are bound by special proteins, designated 1–6. Insulin-like growth factor binding protein-1 (IGFBP-1) is a 25-kDa hydrophobic protein [3]. IGFBP-1 is synthetized and secreted by human adult and fetal hepatocytes, ovarian granulosa cells and decidual cells [4–6]. In pregnant women’s serum the concentration of IGFBP-1 increases during gestation [3]. It is also the major protein of the amniotic fluid [7]. Decidua and liver secrete predominantly phosphorylated forms of IGFBP-1, while in the amniotic fluid non-phosphorylated or less phosphorylated isoforms are present [7]. The phosphorylated form is produced mainly by human decidual cells and is present between the chorion and decidua. As the cervix matures and the labor approaches, the chorion and decidua detach and phosphorylated IGFBP-1 (phIGFBP-1) gets into the cervical secretion, thus its presence reflects decidual activation and dilatation of the internal os. Kekki et al. reported that phIGFBP-1 presence in cervical secretion is a predictor of preterm delivery [8]. This relationship was also confirmed by other authors [9–15]. Cervival evaluation with Bishop score and ultrasound measurement of cervical canal length are usually used to predict the spontaneous onset of labor or successful vaginal delivery. While the phIGFBP-1 presence in cervical secretion in predicting preterm labor is well established, data regarding its similar role in predicting term or post-term delivery are limited. Nuutila et al. [3] confirmed that the IGFBP-1 concentration was higher in clinically ripe than unripe cervices secretions. Basing on the same theoretical premises, the study evaluating IGFBP-1 in prediction of spontaneous and induced vaginal delivery in term and post-term pregnancies was carried out.

The aim of the study was to estimate whether insulin-like growth factor binding protein-1 (IGFBP-1) presence evaluation in cervical secretion in term and post-term pregnancies can predict spontaneous onset of labor or successful vaginal delivery in induced labors in comparison to ultrasound cervical length measurement and Bishop score.

## Materials and methods

The prospective cohort study was carried out at the 1st Department of Obstetrics and Gynecology, Medical University of Warsaw, between January 2013 and January 2014. The studied group consisted of 167 pregnant women admitted for routine induction of labor 7–10 days post-term. Patients with elective indications for cesarean section were excluded from the study. All the pregnancies were dated according to the last menstrual period and verified by the crown-rump length measured in the first trimester. None of the patients had regular uterine contractions or rupture of membranes on admission. Prior to any examination, cervical swab samples were collected with a special kit and the presence of phIGFBP-1 was evaluated by an immunoenzymometric assay (Actim Partus Medix Biochemica). The test is positive when phIGFBP-1 reaches the concentration of 10 μg/L. Cervical secretion samples were obtained by inserting a sterile swab into the cervical canal for 15 s. The swab was afterwards swirled in the extraction solution for 10 s. After the extraction the test dipstick was placed in the solution. The test was checked after 5 min: if the two lines (control line and test line) appeared, the test was considered positive. If only test line appeared, the test was negative. There were no cases of failed tests with no test line visible. Ultrasound assessment of cervical length and Bishop score evaluation of the cervix were carried out afterwards. Cervical length was measured with ultrasound transvaginal 7.5 MHz probe placed in the anterior fornix with empty bladder and the linear distance between calipers placed at the internal and external os was taken into account. All the patients had a routine ultrasound scan performed, assessing fetal biometry. All the women were qualified for labor pre-induction with Foley catheter inserted through the cervical canal and the balloon was filled with 80 ml of 0.9 % NaCl above the internal os. After 24 h the catheter was removed and subsequently labor induction was carried out if spontaneous uterine contractions had not occurred. Following the catheter removal another Bishop score evaluation of the cervix was performed. Routine labor induction was performed with intravenous infusion of oxytocin (5 units of oxytocin in 50 ml 0.9 % NaCl with increasing flow from 1.2 to 6.8 ml/h every 20 min till regular uterine contractions occurred, then the adequate flow was provided) and amniotomy when regular uterine contractions were observed. During the first stage of labor epidural analgesia was available for all the patients upon request when dilatation of the cervix reached 4 cm. Spontaneous onset of labor within 24 h was the primary outcome and successful vaginal delivery was the secondary outcome of the study. A comparison of efficacy for the three analyzed methods (physical examination of the cervix according to Bishop score, ultrasound measurement of the cervical canal and IGFBP-1 presence evaluation in the cervical secretion) in predicting the primary and the secondary outcome was performed. Neonatal birth weight and general condition according to Apgar score were also analyzed.

The study obtained the approval of the Ethics Committee of Medical University of Warsaw (number KB/209/2013) and was conducted according to the Declaration of Helsinki.

Statistical analysis was performed with Mann–Whitney *U* test for continuous variables and Chi-squared test for categorical variables. Cut-off points were estimated on the basis of ROC curves. Every analyzed test was characterized by sensitivity, specificity, positive predictive value, negative predictive value, positive likelihood ratio and negative likelihood ratio. A Kaplan–Meier curve was used to visualize time courses. Logistic regression analysis was performed to investigate the impact of individual factors on primary and secondary outcome. Statistica 10.0 was used for statistical analyses. *p* values <0.05 were considered significant and all tests were two-tailed.

## Results

In five patients, phIGFBP-1 results and two ultrasound cervical measurements were missing and they were excluded from the study. Finally 160 women were available for the analysis. In 52 cases spontaneous regular uterine contractions appeared within 24 h of labor pre-induction (32.5 %). 42 of those patients delivered vaginally (80.8 %). In 108 cases labor induction was performed. After Foley catheter removal Bishop score was 9.3 on average (SD 1.4, 95 % CI 1.2–1.7), which was statistically higher than Bishop score assessed on admission (5.3, SD 2.4, 95 % CI 2.1–1.7; *p* < 0.01). Patients with spontaneous onset of labor had statistically lower body mass index (BMI) (average 27, SD 3.6, 95 % CI 26–28 vs average 29, SD 3.8, 95 % CI 28.3–29.8; *p* < 0.001) and had shorter cervix in ultrasound on admission (average 20.5 mm, SD 9, 95 % CI 18–23 vs 25.3 mm, SD 8.2, 95 % CI 23.7–26.8; *p* < 0.001). There were no statistically significant differences regarding age, parity, weight gain during pregnancy and days post-term between the groups of spontaneous onset of labor and labor induction.

In the nulliparous group (120 patients) spontaneous regular uterine contractions appeared in 35 % (42 women) and 55.8 % delivered vaginally (67 women). In the multiparous group the spontaneous onset of labor within 24 h appeared in 25 % of cases (10 women). All the multiparous patients delivered vaginally (40 women).

108 of all women delivered vaginally (67.5 %), 52 had cesarean section (32.5 %) and there were no cases of instrumental vaginal deliveries (vacuum extractor or forceps). Women, who delivered vaginally, were significantly older (average 31.6 years, SD 5.1, 95 % CI 4.5–5.9 vs 28.8 years, SD 3.1, 95 % CI 2.6–3.8; *p* < 0.001) and had lower BMI (average 27.9, SD 3.5, 95 % CI 27.2–28.6 vs 29.4, SD 4.3, 95 % CI 28.2–30.5; *p* = 0.003), higher initial Bishop score assessment (average 5.6, SD 2.2, 95 % CI 5.2–6 vs 4.7, SD 2.6, 95 % CI 4–5.4; *p* = 0.017) and shorter cervix on ultrasound examination at the time of admission (average 21.5 mm, SD 8.3, 95 % CI 19.9–23 vs 28.3 mm, SD 7.7, 95 % CI 26.2–30.4; *p* < 0.001). There were no cesarean sections performed among patients, who had previously delivered vaginally. There were no statistically significant differences in IGFBP-1 presence in cervical secretion between the groups of vaginal and cesarean delivery (66.67 vs 51.92 %; *p* = 0.07). No statistically significant differences regarding weight gain during pregnancy and days post-term were observed.

In the groups of vaginal and cesarean delivery comparable rates of patients were administered epidural analgesia during the first stage of labor (61.1 vs 50 %; *p* > 0.05). The most common indications for cesarean section were cervical dystocia (59.3 %) and fetal distress (37 %). There were no differences in neonatal birth weight (average 3,543 g, SD 378, 95 % CI 3,471–3,615 vs 3,549 g, SD 395, 95 % CI 3,441–3,657; *p* > 0.05) and general condition according to Apgar score (median 10 vs 10; *p* > 0.05) between the groups. 91.4 % of neonates were born in good general condition (1st min Apgar score).

**Fig. 1:**
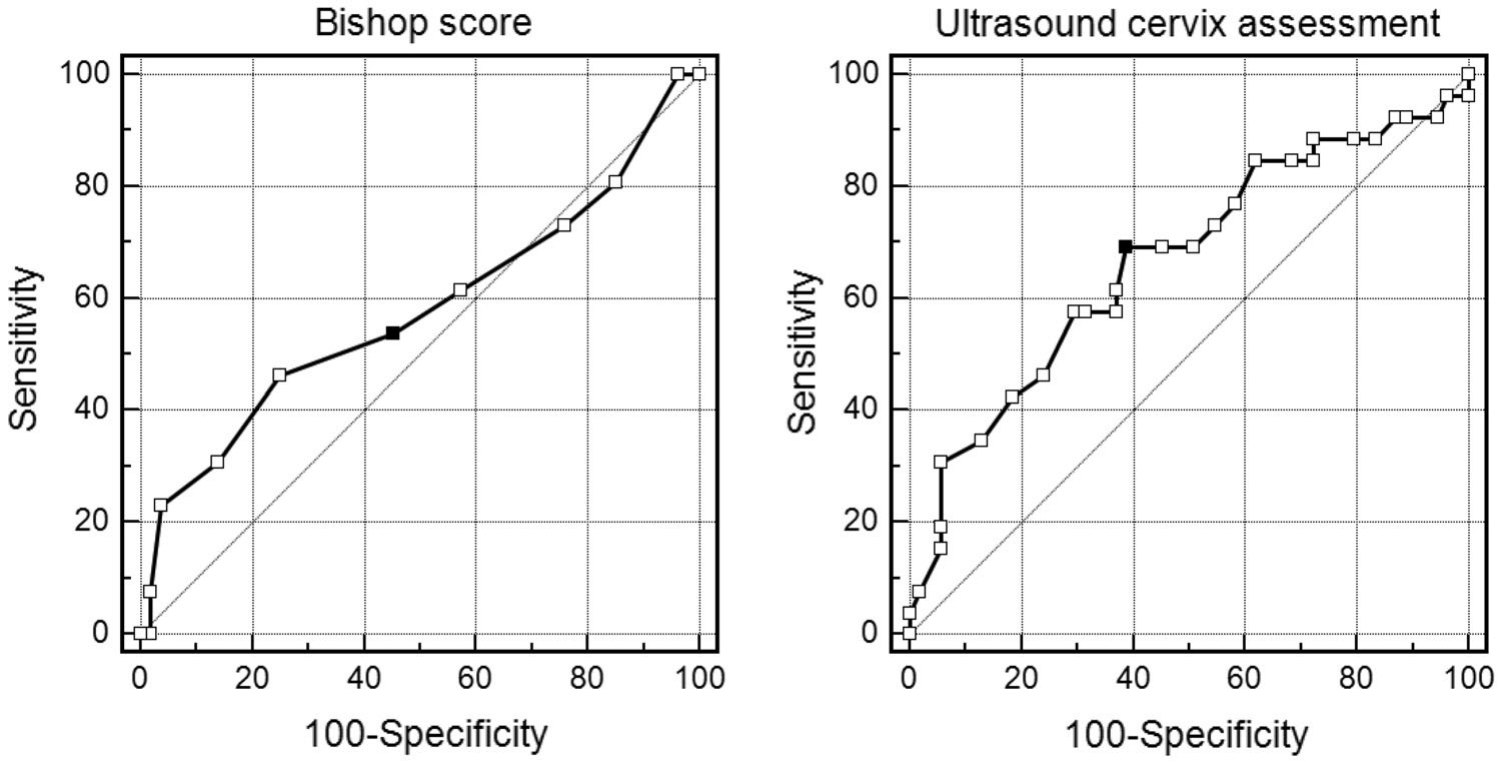
ROC curves used to estimate the cut-off points for the Bishop score (*black dot* is 6 points) and ultrasound cervical measurement (*black dot* is 22 mm) for spontaneous onset of labor prediction

**Fig. 2:**
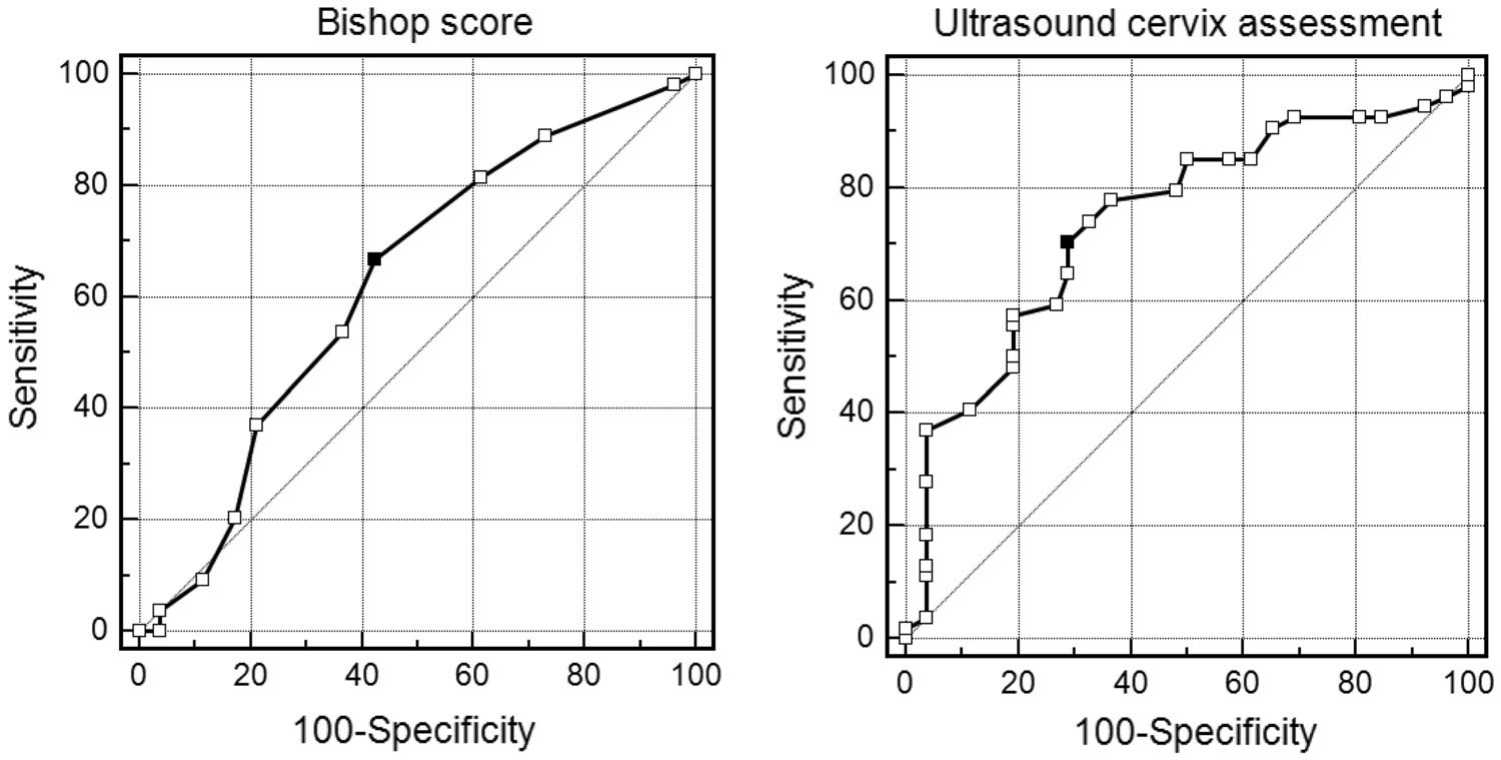
ROC curves used to estimate the cut-off points for the Bishop score (*black dot* is 5 points) and ultrasound cervical measurement (*black dot* is 25 mm) for successful vaginal delivery prediction

The cut-off points for ultrasound cervix assessment and Bishop score were designated on the basis of ROC curves. The cut-off points to predict spontaneous onset of labor were six or more for Bishop score and 22 mm or less for ultrasound cervical length, while for predicting vaginal birth they were, respectively, 5 or more and 25 mm or less. The ROC curves are presented in Figs. 1 and 2.

**Fig. 3:**
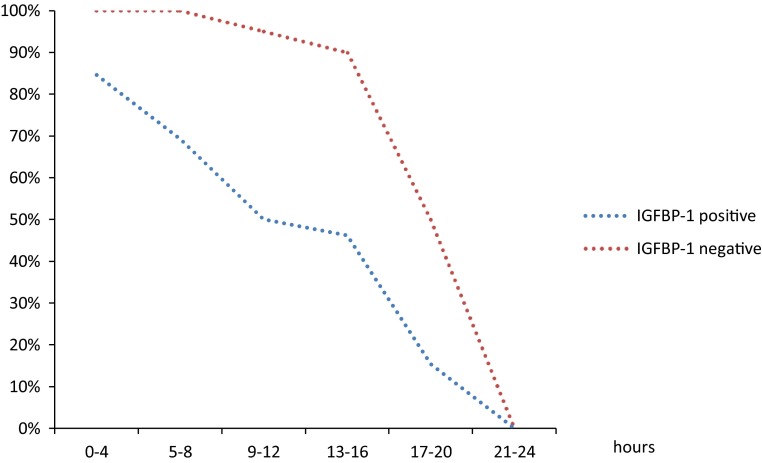
The Kaplan–Meier curves showing the percentage of patients with no active phase of labor after preinduction in time

phIGFBP-1 test predicted spontaneous onset of regular uterine contractions with the sensitivity of 0.69 and specificity of 0.42 (Table 1). Ultrasound measurement of cervical length had the same sensitivity (0.69) with not significantly higher specificity (0.61; *p* = 0.06). Bishop score had slightly lower sensitivity (0.53; *p* > 0.05) with the specificity of 0.55 (*p* > 0.05) in the prediction of spontaneous onset of labor. The time from preinduction to spontaneous onset of delivery was significantly shorter in women with positive phIGFBP-1 test (13.65 ± 6.7 vs 20.75 ± 2.6 h; *p* = 0.006). The Kaplan–Meier curves for the time to spontaneous delivery onset are presented in Fig. 3.

**Table 1:** The characteristics for phIGFBP-1, Bishop score and ultrasound cervix assessment in predicting spontaneous onset of labor and successful vaginal delivery

	Sensitivity (95 % CI)	Specificity (95 % CI)	Positive predictive value (PPV) (95 % CI)	Negative predictive value (NPV) (95 % CI)	Positive likelihood ratio (+LR) (95 % CI)	Negative likelihood ratio (−LR) (95 % CI)
Primary outcome—spontaneous onset of labor						
IGFBP-1 presence	0.69 (0.57–0.8)	0.42 (0.36–0.47)	0.36 (0.3–0.42)	0.74 (0.64–0.83)	1.19 (0.9–1.5)	0.74 (0.43–1.18)
Bishop score (≥6)	0.53 (0.42–0.66)	0.55 (0.49–0.61)	0.37 (0.29–0.45)	0.71 (0.64–0.78)	1.2 (0.83–1.67)	0.84 (0.57–1.18)
Ultrasound cervix assessment (≤22 mm)	0.69 (0.57–0.8)	0.61 (0.55–0.66)	0.46 (0.38–0.53)	0.81 (0.73–0.87)	1.78 (1.28–2.35)	0.5 (0.31–0.77)
Secondary outcome—successful vaginal delivery						
IGFBP-1 presence	0.67 (0.61–0.72)	0.48 (0.36–0.6)	0.73 (0.67–0.79)	0.41 (0.31–0.51)	1.28 (0.96–1.89)	0.69 (0.46–1.07)
Bishop score (≥5)	0.67 (0.61–0.72)	0.58 (0.46–0.69)	0.77 (0.7–0.83)	0.46 (0.36–0.54)	1.58 (1.12–2.32)	0.58 (0.41–0.86)
Ultrasound cervix assessment (≤25 mm)	0.7 (0.65–0.75)	0.71 (0.59–0.81)	0.84 (0.77–0.89)	0.54 (0.45–0.61)	2.44 (1.59–3.98)	0.42 (0.31–0.6)

The presence of phIGFBP-1 predicted successful vaginal delivery with the sensitivity of 0.67 and the specificity of 0.48 (Table 1). Bishop score had the same sensitivity (0.67), but higher specificity (0.58; *p* > 0.05) and ultrasound cervix assessment had higher sensitivity (0.7; *p* > 0.05) and significantly higher specificity (0.71; *p* = 0.03) in predicting successful vaginal birth.

The further data analysis concerning patients’ parity was carried out. The studied group consisted of 120 primiparas (75 %). In the nulliparous group the sensitivity of phIGFBP-1 test in predicting spontaneous delivery onset was higher than the Bishop score (0.67 vs 0.43; *p* = 0.048) and not significantly lower than cervical length assessment (0.71; *p* > 0.05) (Table 2). Both Bishop score and ultrasound cervix measurement had higher specificity (Bishop score 0.63 vs phIGFBP-1 0.4; *p* = 0.006 and ultrasound cervical length 0.67 vs phIGFBP-1 0.4; *p* = 0.001) in the prediction of spontaneous delivery onset. In the multiparous women group the presence of phIGFBP-1 in cervical secretion predicted spontaneous onset of labor with high sensitivity (0.8, specificity 0.47). Bishop score and ultrasound cervical length measurement did not differ significantly when sensitivity and specificity were compared. phIGFBP-1 test’s sensitivity was higher than Bishop score in predicting successful vaginal labor in nulliparous women (0.71 vs 0.56), but lower than ultrasound cervical assessment (0.77), however, the differences were not significant. phIGFBP-1 test had lowest specificity (0.48 vs Bishop score: 0.58; *p* > 0.05; vs cervical length: 0.71; *p* = 0.03). As all the multiparous patients delivered vaginally, the statistical analysis of vaginal birth prediction was not carried out.

**Table 2:** The characteristics for phIGFBP-1, Bishop score and ultrasound cervix assessment in predicting spontaneous onset of labor and successful vaginal delivery in nulliparous and parous patients

	Sensitivity (95 % CI)	Specificity (95 % CI)	Positive predictive value (PPV) (95 % CI)	Negative predictive value (NPV) (95 % CI)	Positive likelihood ratio (+LR) (95 % CI)	Negative likelihood ratio (−LR) (95 % CI)
Primary outcome—spontaneous onset of labor						
Nulliparous						
IGFBP-1 presence	0.67 (0.54–0.79)	0.4 (0.33–0.46)	0.37 (0.3–0.44)	0.69 (0.57–0.8)	1.11 (0.8–1.46)	0.84 (0.47–1.42)
Bishop score (≥6)	0.43 (0.3–0.56)	0.63 (0.56–0.7)	0.38 (0.27–0.5)	0.67 (0.6–0.75)	1.15 (0.69–1.85)	0.91 (0.63–1.25)
Ultrasound cervix assessment (≤22 mm)	0.71 (0.58–0.82)	0.67 (0.6–0.73)	0.54 (0.44–0.62)	0.81 (0.73–0.88)	2.14 (1.44–3)	0.43 (0.24–0.7)
Parous						
IGFBP-1 presence	0.8 (0.48–0.96)	0.47 (0.36–0.52)	0.33 (0.2–0.4)	0.88 (0.68–0.98)	1.5 (0.76–2)	0.43 (0.07–1.43)
Bishop score (≥6)	0.9 (0.63–1)	0.31 (0.21–0.34)	0.33 (0.23–0.37)	0.9 (0.6–1)	1.32 (0.8–1.5)	0.29 (0.01–1.78)
Ultrasound cervix assessment (≤22 mm)	0.6 (0.3–0.85)	0.47 (0.37–0.55)	0.27 (0.14–0.39)	0.78 (0.61–0.92)	1.13 (0.48–0.9)	0.86 (0.27–1.9)
Secondary outcome—successful vaginal delivery						
Nulliparous						
IGFBP-1 presence	0.71 (0.62–0.78)	0.48 (0.37–0.58)	0.64 (0.57–0.71)	0.56 (0.43–0.67)	1.36 (0.99–1.88)	0.61 (0.37–1)
Bishop score (≥5)	0.56 (0.47–0.64)	0.58 (0.47–0.68)	0.63 (0.54–0.73)	0.5 (0.4–0.59)	1.32 (0.89–2)	0.77 (0.53–1.13)
Ultrasound cervix assessment (≤25 mm)	0.77 (0.68–0.83)	0.71 (0.6–0.8)	0.78 (0.69–0.85)	0.7 (0.59–0.79)	2.65 (1.72–4.17)	0.33 (0.21–0.53)

Multiple regression analysis regarding age, parity, BMI, neonatal birth weight, Bishop score, phIGFBP-1 presence and cervical length was performed. For the spontaneous onset of labor within 24 h significant odds ratios were shown only for BMI (OR 0.2, 95 % CI 0.06–0.6; *p* = 0.005) and cervical length assessment (OR 3.69, 95 % CI 1.6–8.55; *p* = 0.002). Odds ratios calculated for the successful vaginal birth were significant for age (OR 1.18, 95 % CI 1.07–1.3; *p* < 0.001) and ultrasound cervical length assessment (OR 5.16, 95 % CI 2.28–11.67; *p* < 0.001).

## Discussion

Post-term pregnancies are associated with adverse maternal and neonatal outcome. In Danish birth-registry study, increased rates of multiple maternal and perinatal complications were found in singleton pregnancies of at least 42 weeks’ gestation [16]. The risk of fetal death in pregnancy beyond 42 weeks of gestation is twice as high as at term and increases sixfold and higher at 43 weeks [17]. Induction of labor is therefore a common procedure. It is performed in about 15–25 % of all pregnancies [18]. According to the Cochrane review, induction of labor in post-term pregnancy decreases the risk of perinatal death (RR 0.31) [19]. Although post-term gestation is defined as a pregnancy of 42 weeks or more, several large multicenter randomized studies regarding the management of pregnancy beyond 40 weeks of gestation reported favorable outcomes with routine induction as early as the beginning of 41 weeks [20–22]. Although there is no sufficient data, management in low-risk post-term pregnancy is better: expectant or active, many societies of obstetricians and gynecologists recommend active management for low-risk women at 41 completed weeks. In cases of unfavorable cervix several cervical ripening methods are available, like prostaglandin preparations or labor preinduction with Foley catheter. The risk of cesarean section during labor induction is increased in post-term pregnancy and varies between 10 and 37 % [19, 23–28]. In the presented study group 32.5 % of patients had cesarean section performed. The main indication for operative delivery during labor induction is cervical dystocia. According to our data almost 60 % of cesarean sections were performed due to this indication. Cesarean section during labor induction may be associated with higher risk of complications, especially excessive blood loss. A sensitive diagnostic tool to predict spontaneous onset of labor and successful vaginal delivery in labor induction in term or post-term pregnancies might be very helpful in making clinical decisions regarding the adequate management—expectant or active.

We found phIGFBP-1 test to be a predictor of spontaneous onset of labor and successful vaginal delivery in post-term pregnancies. It’s sensitivity did not differ significantly from Bishop score and ultrasound cervix assessment. As the ultrasound measurement of cervical length had significantly higher specificity in predicting both outcomes, it seems to be the best predictor of spontaneous labor onset and successful vaginal delivery. Women with the positive phIGFBP-1 test also developed regular uterine contractions earlier after labor preinduction. In the subgroup of multiparous patients all three tests had comparable sensitivity and specificity for predicting both outcomes. In the nulliparous group phIGFBP-1 presence predicted spontaneous delivery onset with significantly higher sensitivity than Bishop score, which is the most common tool for the evaluation of cervix readiness for delivery and the most common predictor of successful vaginal birth [27]. The evaluation of Bishop score is also subjective and may have a significant inter-investigator variability. The phIGFBP-1 test is the most objective method of all analyzed tools.

According to Dőgl et al. [29] IGFBP-1 has a lower sensitivity (0.45) and higher specificity (0.80) in comparison to our data. Characteristics of Bishop score were also different (sensitivity 0.24, specificity 0.92), while the ultrasound cervical length assessment was similar (sensitivity 0.67, specificity 0.58). The observed difference may be the consequence of different cut-off points for Bishop score used in our study and by Dőgl (≥5 points without a cut-off evaluation).

The adequate test for successful labor induction in post-term pregnancy is needed. We found that phIGFBP-1 presence in cervical secretion is associated with spontaneous onset of labor and successful vaginal birth. So far mostly Bishop score and cervical length assessment have been analyzed in the literature as the tools for predicting the above. Strobel et al. [30] found both of them to predict spontaneous onset of labor within 24 and 48 h in term women. Bishop score was an independent predictor in nulliparous women within 96 h. In parous patients none of them could be used to predict labor in 96 h. According to Rozenberg et al. [31] Bishop score and cervical length were closely associated with spontaneous onset of delivery within 7 days period. Both Bishop score and cervical length were strong predictors of delivery within 48 h according to Rovas et al. [32]. Also Vankayalapati et al. [33] suggested that ultrasound assessment of cervical length is a significant independent predictor of the likelihood of the onset of spontaneous delivery in nulliparous women, and of successful vaginal delivery in both nulliparous and parous women with prolonged pregnancy. According to Crane’s metaanalysis all three analyzed factors (ultrasound cervix assessment, Bishop score and fetal fibronectin) predicted successful induction of labor [34]. Cheung et al. [27] found that the only independent predictors of successful labor induction were multiparity, transvaginal cervical length and maternal height <150 cm. Also in our analysis ultrasound measurement of cervical length was a strong predictor of vaginal birth. According to our results phIGFBP-1 may also be the useful predictor of spontaneous onset of labor within 24 h and successful vaginal birth, but it’s sensitivity is comparable with other analyzed tests. In the multiparous subgroup all three tests were comparable, but in the nulliparous group phIGFBP-1 and ultrasound cervix assessment were significantly more sensitive in predicting spontaneous delivery onset and comparable in predicting vaginal birth. Therefore, the low-risk nulliparous women might be the target group for phIGFBP-1 test. Our study, analogously to Cheung et al., does not support the routine screening of phIGFBP-1 in post-term pregnancies [27].

The phIGFBP-1 bedside test is quick and easy to perform. It could be used additionally to Bishop score or ultrasound cervical length to predict successful vaginal delivery in post-term patients. As a supplementary test it could bring additional information for the adequate patients’ qualification for labor induction. It might be useful in deciding between active or expectant management. However, it is not established if the test could be recommended on economic basis. Although its cost effectiveness is yet to be determined, it seems that it could increase the costs of medical management in post-term pregnancy.

## Conclusion

A test for phIGFBP1 presence might be an additional tool for predicting both spontaneous onset of labor and successful vaginal delivery in post-term pregnancies, but our study does not support the routine screening for phIGFBP-1 in prediction of vaginal birth in post-term pregnancies.
